# Supplementary material for: Proteomic atlas of organ vasculopathies triggered by Staphylococcus aureus sepsis
Source: Nat Commun. 2019 Oct 11;10:4656. doi: 10.1038/s41467-019-12672-x (PMC6789120; doi:10.1038/s41467-019-12672-x)
Supplement: Supplementary file 1 — Supplementary Information [file 41467_2019_12672_MOESM1_ESM.pdf]

Supplementary Information for:

"Proteomic atlas of organ vasculopathies triggered by Staphylococcus aureus sepsis"

Alejandro Gómez Toledo et al

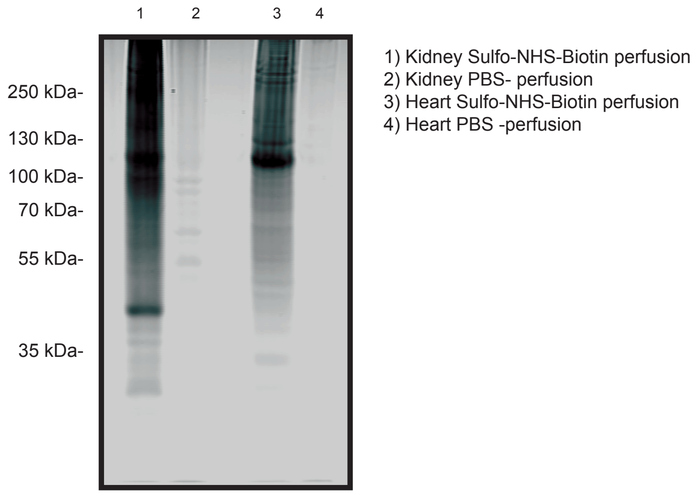

Supplementary Figure 1. Western blotting analysis of organ lysates derived from systemically biotinylated mice. Kidney and heart tissues from animals subjected to perfusion with sulfo-NHS-biotin were homogenized and resolved by SDS-PAGE. Protein signals were detected by incubating with a streptavidin probe conjugated to an infrared dye. Strong streptavidin reactivity was detected in the biotinylated samples (lanes 1 and 3). Only faint signals were observed in control tissue derived from animals perfused with PBS (lanes 2 and 4).

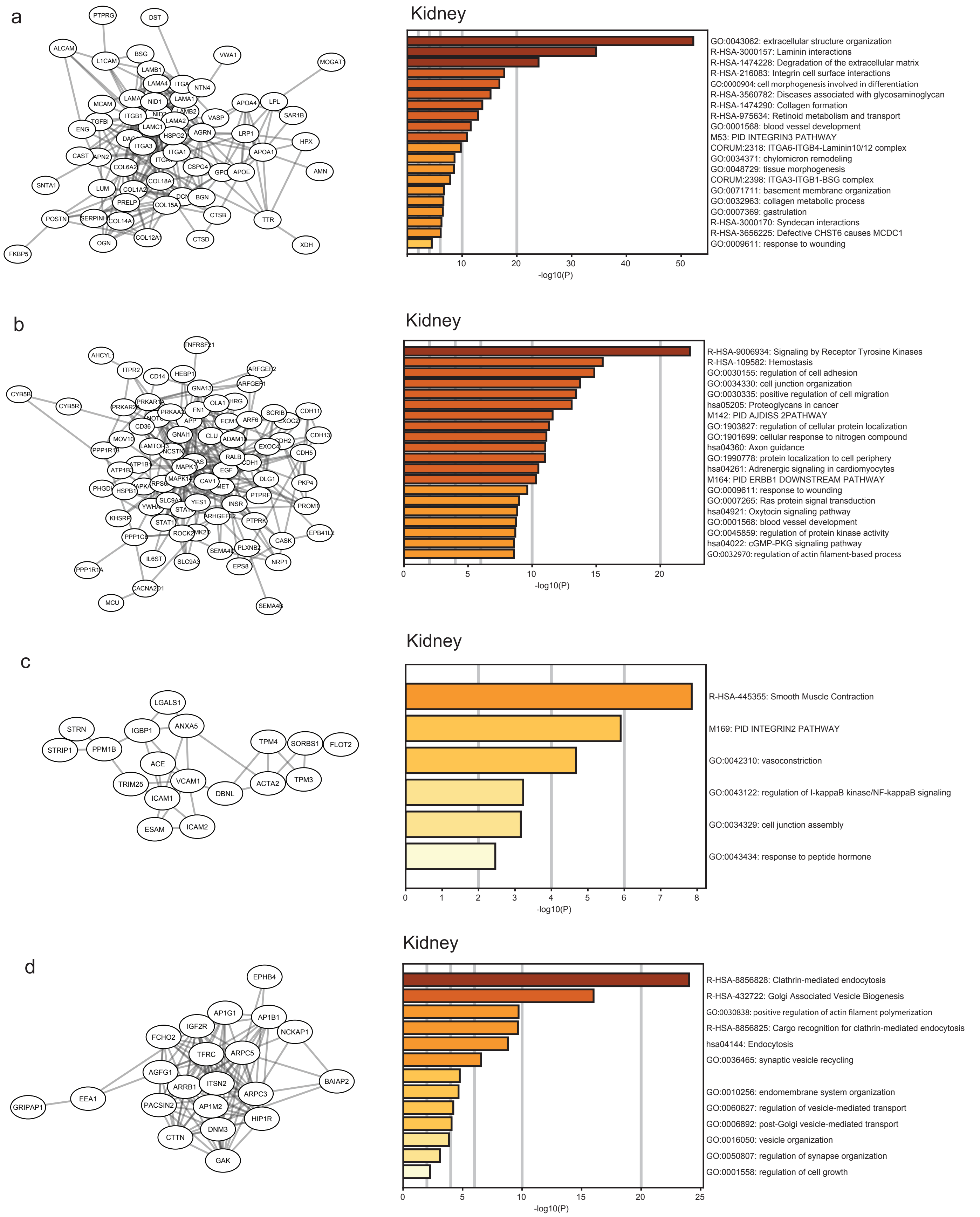
a
Kidney
GO:0043062: extracellular structure organization
R-HSA-3000157: Laminin interactions
R-HSA-1474228: Degradation of the extracellular matrix
R-HSA-216083: Integrin cell surface interactions
GO:0000904: cell morphogenesis involved in differentiation
R-HSA-3560782: Diseases associated with glycosaminoglycan
R-HSA-1474290: Collagen formation
R-HSA-975634: Retinoid metabolism and transport
GO:0001568: blood vessel development
M53: PID INTEGRIN3 PATHWAY
CORUM:2318: ITGA6-ITGB4-Laminin10/12 complex
GO:0034371: chylomicron remodeling
GO:0048729: tissue morphogenesis
CORUM:2398: ITGA3-ITGB1-BSG complex
GO:0071711: basement membrane organization
GO:0032963: collagen metabolic process
GO:0007369: gastrulation
R-HSA-3000170: Syndecan interactions
R-HSA-3656225: Defective CHST6 causes MCDC1
GO:0009611: response to wounding
10
20
30
40
50
-log10(P)
b
Kidney
R-HSA-9006934: Signaling by Receptor Tyrosine Kinases
R-HSA-109582: Hemostasis
GO:0030155: regulation of cell adhesion
GO:0034330: cell junction organization
GO:0030335: positive regulation of cell migration
hsa05205: Proteoglycans in cancer
M142: PID AJDISS 2PATHWAY
GO:1903827: regulation of cellular protein localization
GO:1901699: cellular response to nitrogen compound
hsa04360: Axon guidance
GO:1990778: protein localization to cell periphery
hsa04261: Adrenergic signaling in cardiomyocytes
M164: PID ERBB1 DOWNSTREAM PATHWAY
GO:0009611: response to wounding
GO:0007265: Ras protein signal transduction
hsa04921: Oxytocin signaling pathway
GO:0001568: blood vessel development
GO:0045859: regulation of protein kinase activity
hsa04022: cGMP-PKG signaling pathway
GO:0032970: regulation of actin filament-based process
0
5
10
15
20
-log10(P)
c
LGALS1
STRN
IGBP1
ANXA5
STRIP1
PPM1B
ACE
TPM4
SORBS1
FLOT2
TRIM25
VCAM1
DBNL
ACTA2
TPM3
ICAM1
ESAM
ICAM2
Kidney
R-HSA-445355: Smooth Muscle Contraction
M169: PID INTEGRIN2 PATHWAY
GO:0042310: vasoconstriction
GO:0043122: regulation of I-kappaB kinase/NF-kappaB signaling
GO:0034329: cell junction assembly
GO:0043434: response to peptide hormone
0
1
2
3
4
5
6
7
8
-log10(P)
d
EPHB4
AP1G1
AP1B1
IGF2R
FCHO2
NCKAP1
TFRC
ARPC5
AGFG1
ITSN2
BAIAP2
ARRB1
ARPC3
EEA1
PACSIN2
AP1M2
GRIPAP1
HIP1R
DNM3
CTTN
GAK
Kidney
R-HSA-8856828: Clathrin-mediated endocytosis
R-HSA-432722: Golgi Associated Vesicle Biogenesis
GO:0030838: positive regulation of actin filament polymerization
R-HSA-8856825: Cargo recognition for clathrin-mediated endocytosis
hsa04144: Endocytosis
GO:0036465: synaptic vesicle recycling
GO:0010256: endomembrane system organization
GO:0060627: regulation of vesicle-mediated transport
GO:0006892: post-Golgi vesicle-mediated transport
GO:0016050: vesicle organization
GO:0050807: regulation of synapse organization
GO:0001558: regulation of cell growth
0
5
10
15
20
25
-log10(P)

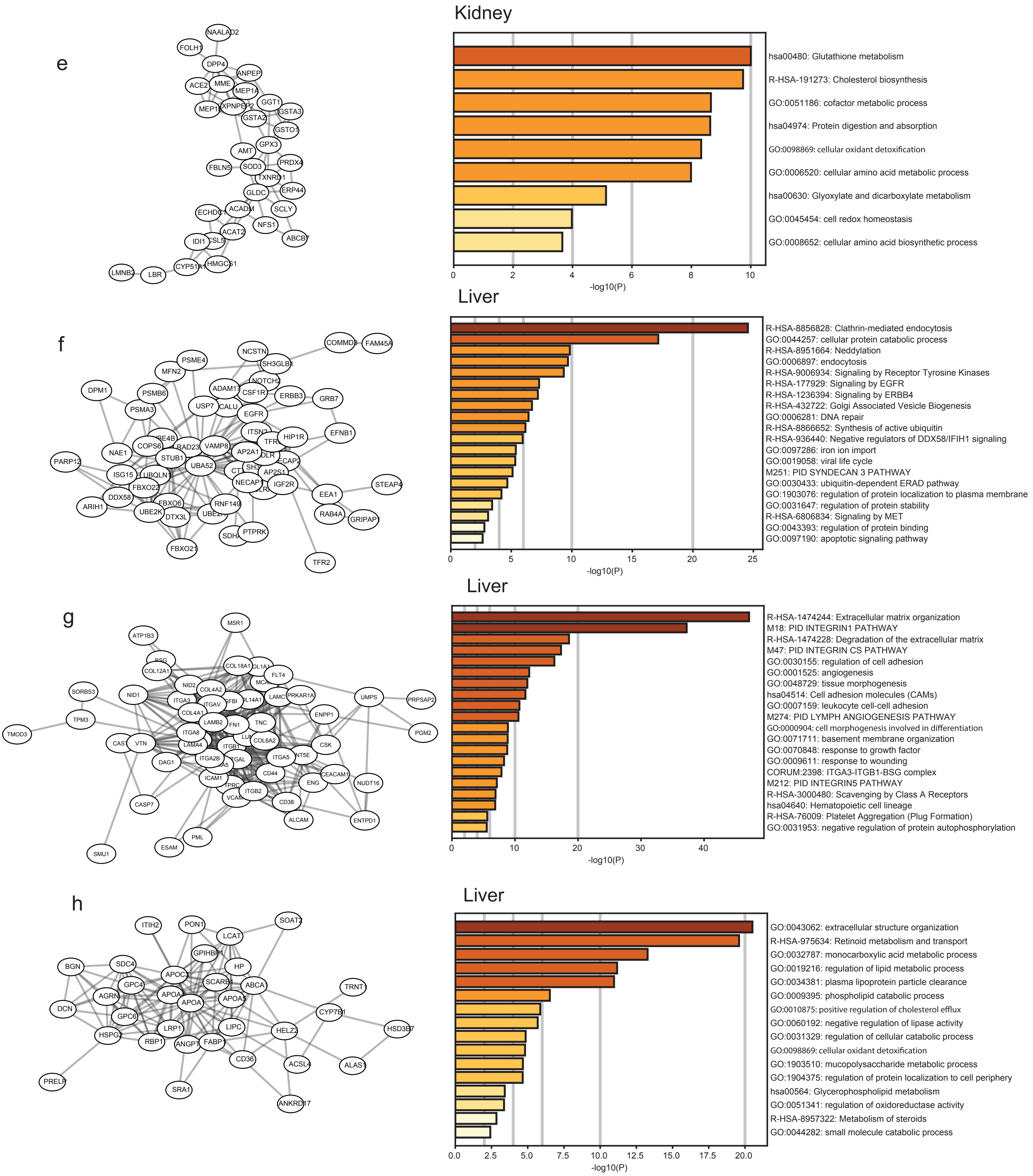

e
Kidney
hsa00480: Glutathione metabolism
R-HSA-191273: Cholesterol biosynthesis
GO:0051186: cofactor metabolic process
hsa04974: Protein digestion and absorption
GO:0098869: cellular oxidant detoxification
GO:0006520: cellular amino acid metabolic process
hsa00630: Glyoxylate and dicarboxylate metabolism
GO:0045454: cell redox homeostasis
GO:0008652: cellular amino acid biosynthetic process
0 2 4 6 8 10
-log10(P)
f
Liver
R-HSA-8856828: Clathrin-mediated endocytosis
GO:0044257: cellular protein catabolic process
R-HSA-8951664: Neddylation
GO:0006897: endocytosis
R-HSA-9006934: Signaling by Receptor Tyrosine Kinases
R-HSA-177929: Signaling by EGFR
R-HSA-1236394: Signaling by ERBB4
R-HSA-432722: Golgi Associated Vesicle Biogenesis
GO:0006281: DNA repair
R-HSA-8866652: Synthesis of active ubiquitin
R-HSA-936440: Negative regulators of DDX58/IFIH1 signaling
GO:0097286: iron ion import
GO:0019058: viral life cycle
M251: PID SYNDECAN 3 PATHWAY
GO:0030433: ubiquitin-dependent ERAD pathway
GO:1903076: regulation of protein localization to plasma membrane
GO:0031647: regulation of protein stability
R-HSA-6806834: Signaling by MET
GO:0043393: regulation of protein binding
GO:0097190: apoptotic signaling pathway
0 5 10 15 20 25
-log10(P)
g
Liver
R-HSA-1474244: Extracellular matrix organization
M18: PID INTEGRIN1 PATHWAY
R-HSA-1474228: Degradation of the extracellular matrix
M47: PID INTEGRIN CS PATHWAY
GO:0030155: regulation of cell adhesion
GO:0001525: angiogenesis
GO:0048729: tissue morphogenesis
hsa04514: Cell adhesion molecules (CAMs)
GO:0007159: leukocyte cell-cell adhesion
M274: PID LYMPH ANGIOGENESIS PATHWAY
GO:0000904: cell morphogenesis involved in differentiation
GO:0071711: basement membrane organization
GO:0070848: response to growth factor
GO:0009611: response to wounding
CORUM:2398: ITGA3-ITGB1-BSG complex
M212: PID INTEGRIN5 PATHWAY
R-HSA-3000480: Scavenging by Class A Receptors
hsa04640: Hematopoietic cell lineage
R-HSA-76009: Platelet Aggregation (Plug Formation)
GO:0031953: negative regulation of protein autophosphorylation
0 10 20 30 40
-log10(P)
h
Liver
GO:0043062: extracellular structure organization
R-HSA-975634: Retinoid metabolism and transport
GO:0032787: monocarboxylic acid metabolic process
GO:0019216: regulation of lipid metabolic process
GO:0034381: plasma lipoprotein particle clearance
GO:0009395: phospholipid catabolic process
GO:0010875: positive regulation of cholesterol efflux
GO:0060192: negative regulation of lipase activity
GO:0031329: regulation of cellular catabolic process
GO:0098869: cellular oxidant detoxification
GO:1903510: mucopolysaccharide metabolic process
GO:1904375: regulation of protein localization to cell periphery
hsa00564: Glycerophospholipid metabolism
GO:0051341: regulation of oxidoreductase activity
R-HSA-8957322: Metabolism of steroids
GO:0044282: small molecule catabolic process
0.0 2.5 5.0 7.5 10.0 12.5 15.0 17.5 20.0
-log10(P)

i

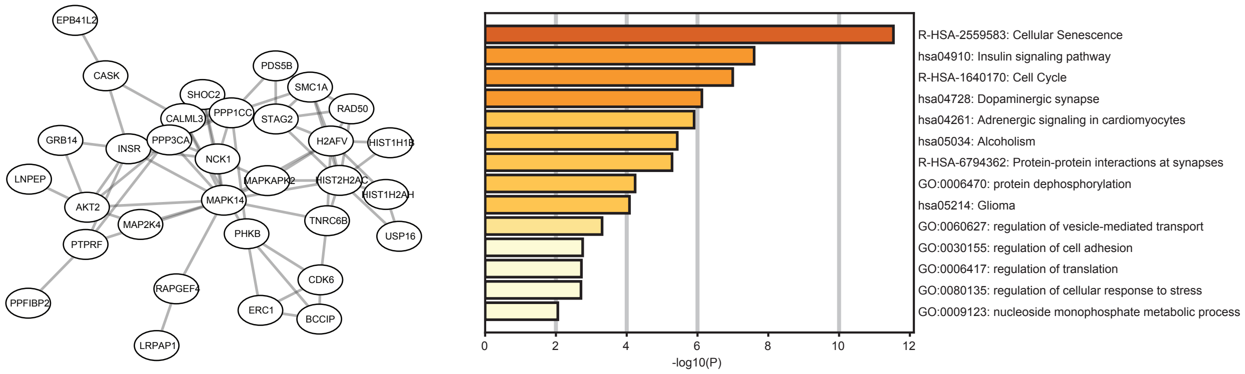

Supplementary Figure 2. Network analysis of systemically biotinylated proteins from liver and kidney tissues. Proteins identified through proteomics analysis were used to generate a protein-protein association network through the STRING database. Protein interactions were limited to high confidence physical or functional associations (association score > 0.7). The final network was subjected to Louvain clustering to identify highly interconnected communities. The identified clusters were further segregated via force-directed visualization algorithms and subjected to functional enrichment analysis using the web-based version of Metascape. Kidney proteins were clustered into 5 different communities (a-e) whereas liver samples were segregated into 4 different cluster (f-i). Some of these clusters were enriched in specific functions and organ specific biological pathways

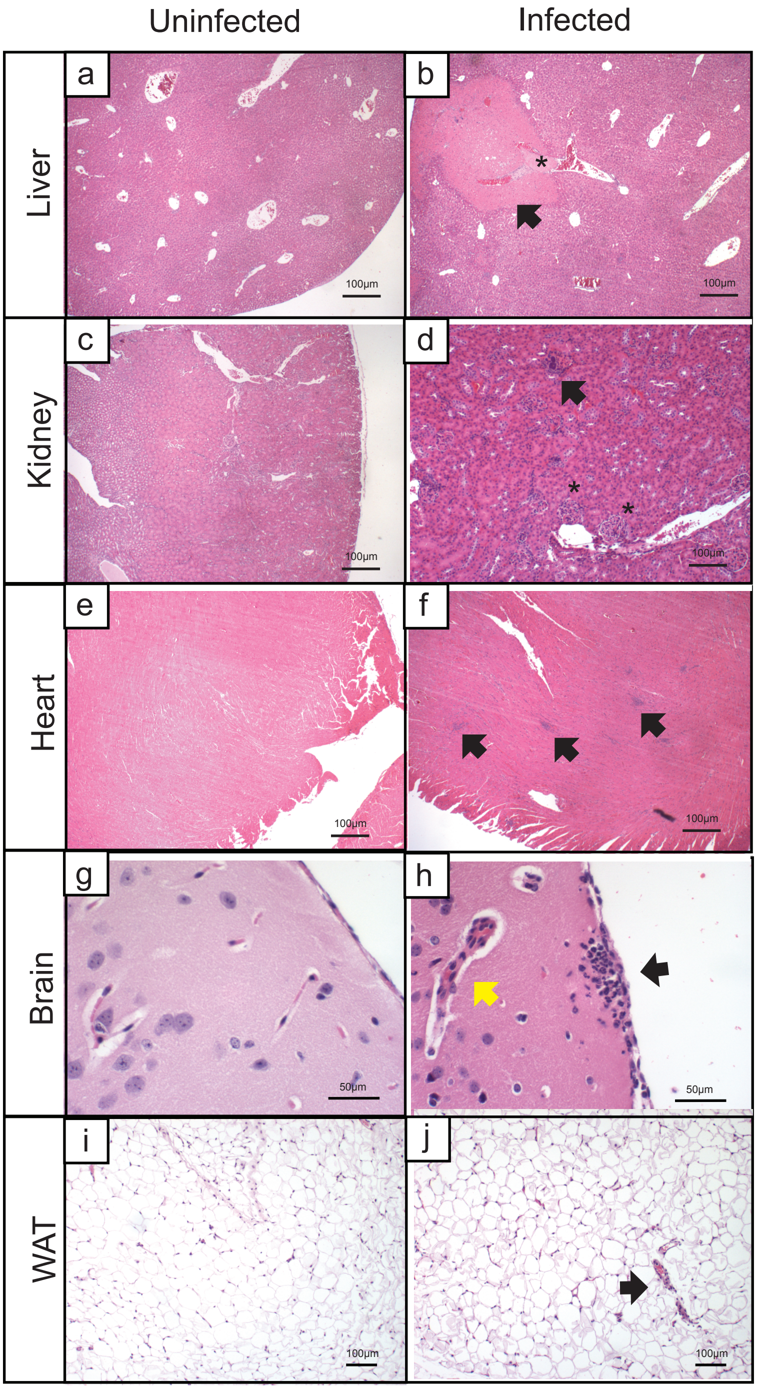

Supplementary Figure 3. Histological analysis of organ pathologies triggered by MRSA-sepsis. Representative hematoxylin and eosin (H&E) stained tissue slides derived from septic animals at 24 hr post-MRSA infection and uninfected controls, reveal multiple signs of inflammation, vasculopathy and bacterial colonization. Liver tissues (a-b) consistently displayed areas of extensive necrosis (black arrow) in the vicinity of large thrombi (asterisk). Signs of leukocyte infiltration and glomerular inflammation (arrows) were observed in the kidney (c-d) together with bacterial outbreaks (arrow). Heart tissues (e-f) showed the presence of multiple neutrophil infiltrates (black arrows). Brain tissues (g-h) did not show any pathological signs although sporadic leukocyte infiltrates (black arrow) and enlargement of brain microvasculature (yellow arrow) was found in some animals. White adipose tissues (i-j) mostly displayed a healthy appearance with rare localized infiltrates (black arrows) in some but not all animals. Scale bars, 50µm, 100µm.

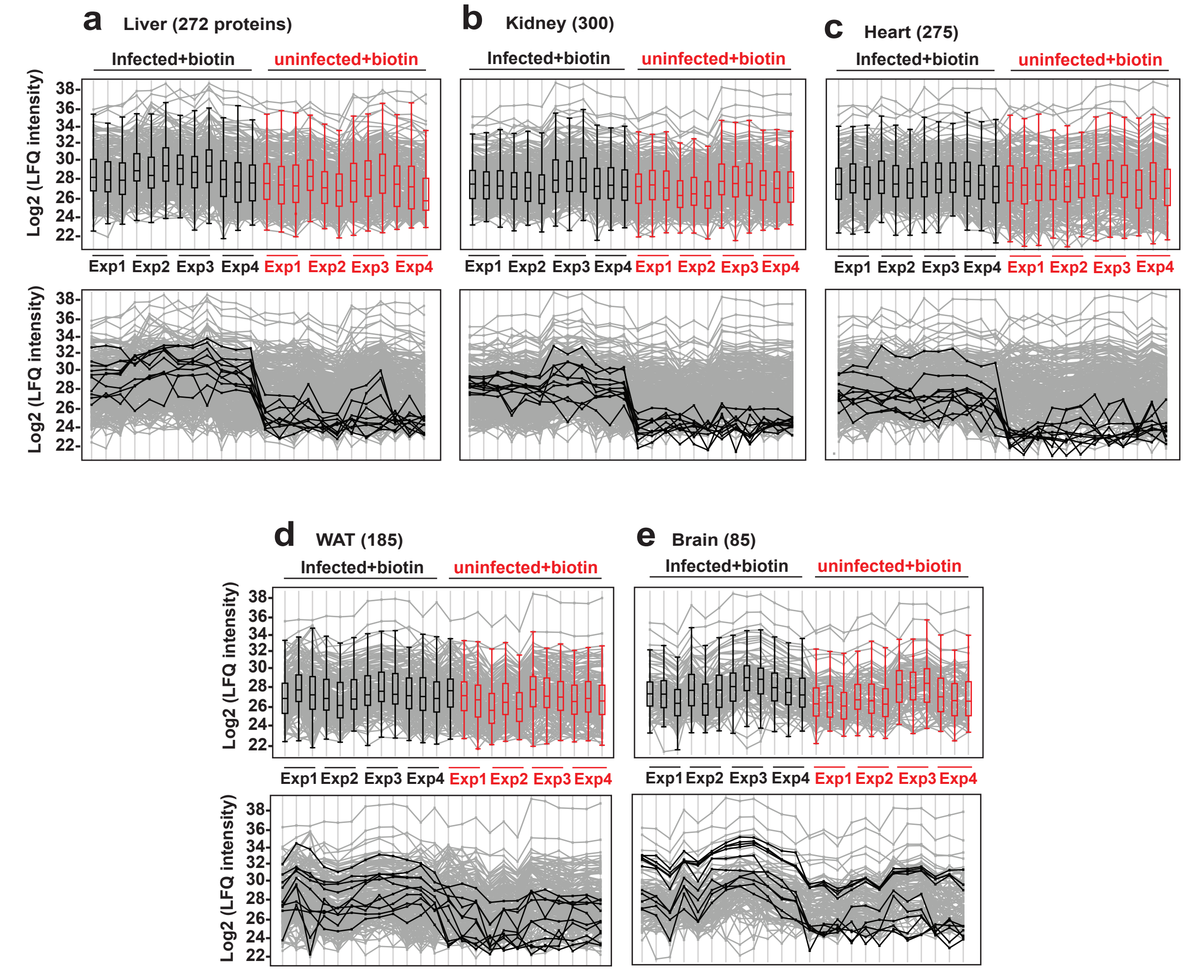

Supplementary Figure 4. Profile plots of the label-free quantification (LFQ) normalized intensities of significant protein targets detected in the organ vasculatures. Combined proteomics datasets of MRSA infected (n=12) and uninfected (n=12) biological replicates of liver (a), kidney (b), heart (c), WAT (d) and brain (e) show that the proteome changes detected by the method encompassed a broad dynamic range. There was a notable intrassay (within the same group) and interassay (within experiments) variability in the LFQ-values (4a-e, upper panels). However, plotting the top 10 differential proteins identified in each tissue makes evident that the changes during infection largely exceed the experimental error since the method is still capable of differentiating between infected and uninfected samples (4a-e, lower panels).

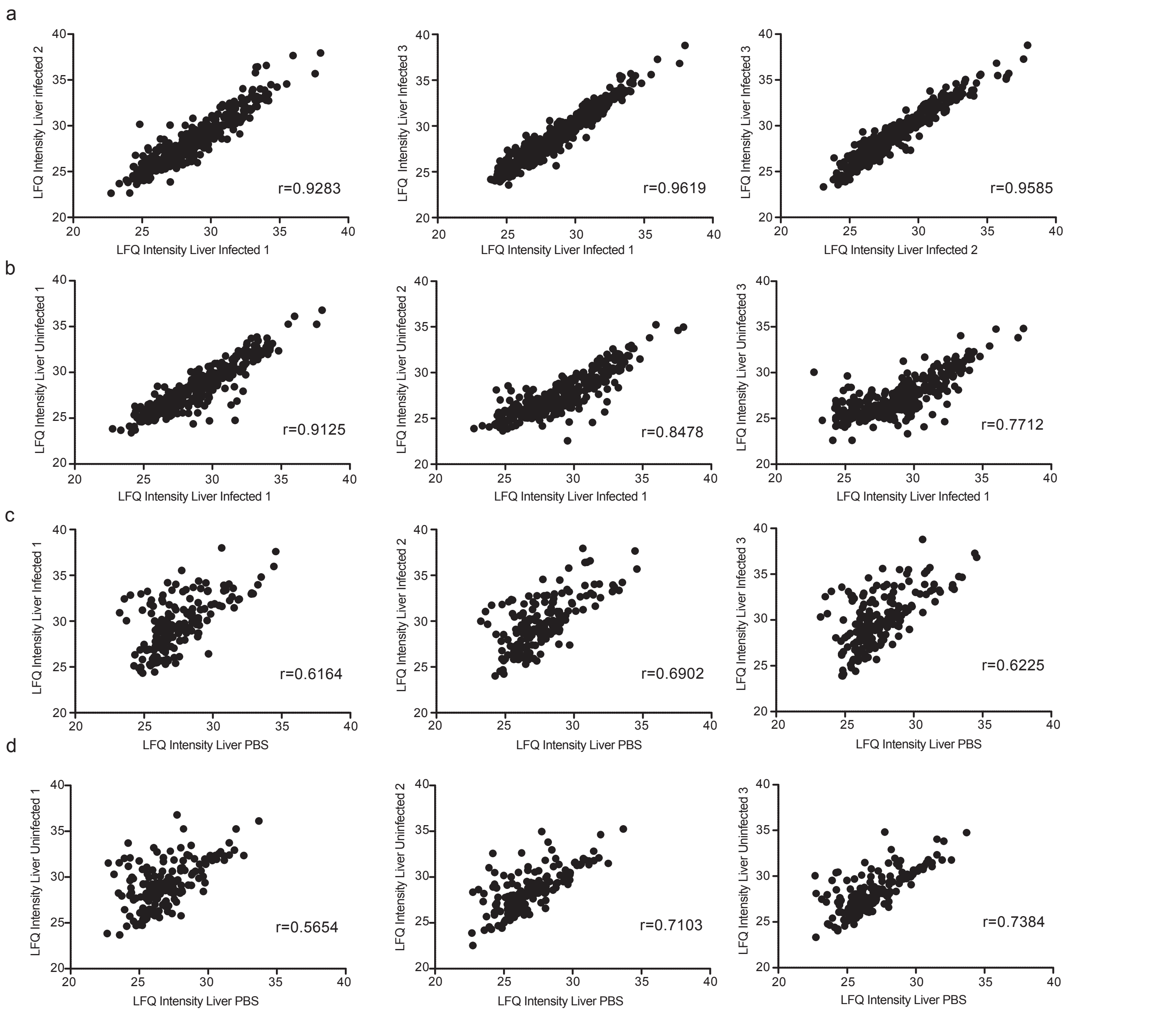

Supplementary Figure 5. Multiple correlation plots of the label-free quantification intensities across liver samples analyzed in the same individual experiment. Pearson correlation coefficients were derived from the plots and compared across biological replicates of infected, uninfected and PBS-control liver tissues. Infected replicates (a) showed high Pearson correlation coefficients, whereas correlations were decreases when comparing infected vs uninfected (b). Even lower correlations were observed when comparing labeled samples with the PBS controls independently of their infection status (c-d).

Supplementary Figure.6

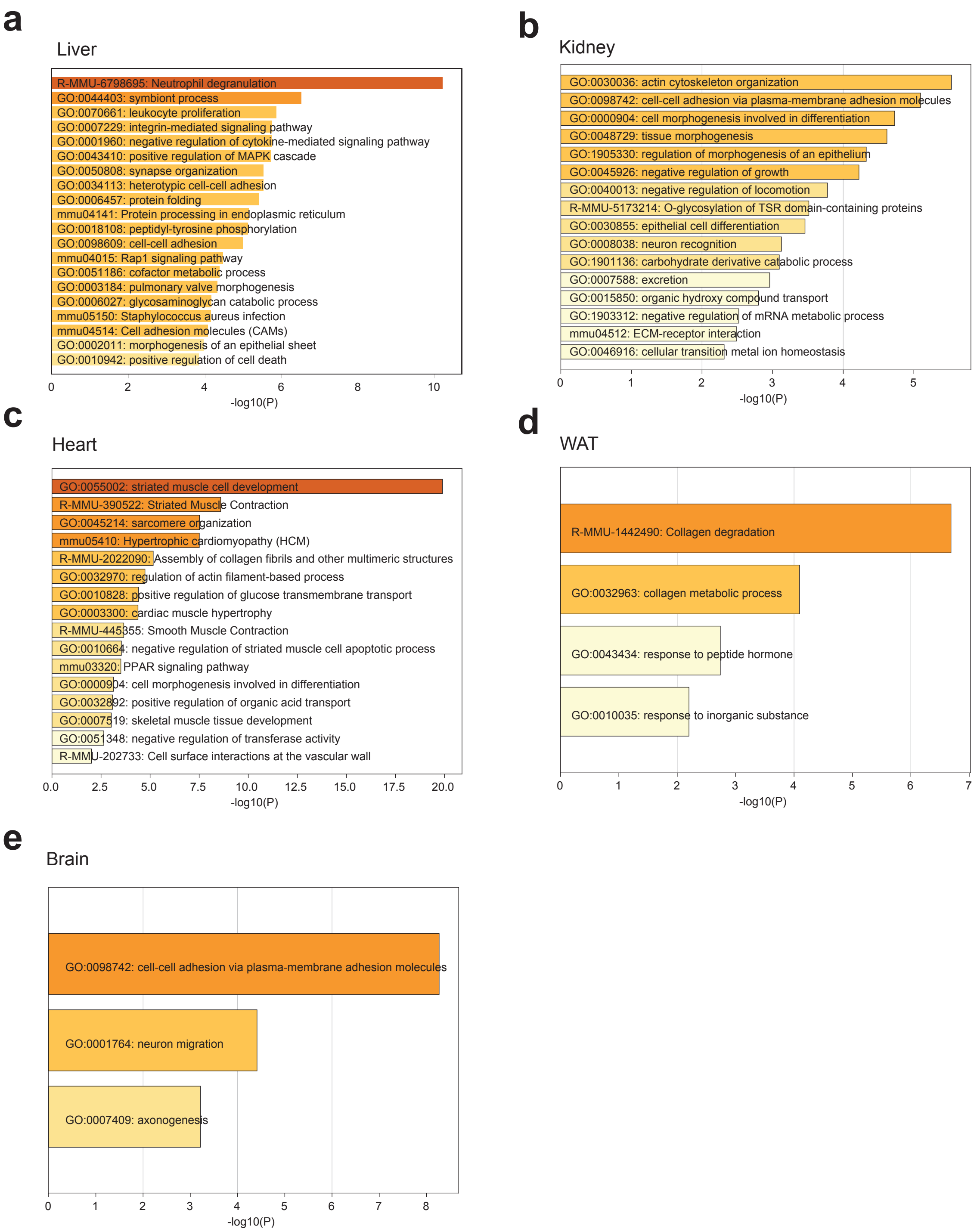

Supplementary Figure 6. Proteomics and functional enrichment analysis of the vascular cell surface proteome identified multiple shared and organ-restricted biological pathways dysregulated during sepsis. All significantly changing proteins across liver (a), kidney (b), heart (c), brain (d) and white adipose tissue (WAT) (e) were analyzed by Metascape resulting in enrichment in particular biological pathways.
