# Supplementary material for: Proteomic atlas of organ vasculopathies triggered by Staphylococcus aureus sepsis
Source: Nat Commun. 2019 Oct 11;10:4656. doi: 10.1038/s41467-019-12672-x (PMC6789120; doi:10.1038/s41467-019-12672-x)
Supplement: Supplementary file 3 — Description of Additional Supplementary Files [file 41467_2019_12672_MOESM3_ESM.docx]

**Description of Supplementary Files**

**File Name:** Supplementary Data 1

**Description:** MaxQuant output of the protein and peptide identifications obtained from 2DLC-MS/MS analysis of biotinylated murine Liver and Kidney samples.

**File Name:** Supplementary Data 2

**Description:** Label-free quantification of proteome changes induced by MRSA-sepsis across the vasculatures of Liver, Kidney, Heart, Brain and White Adipose Tissue (WAT) samples.

**File Name:** Supplementary Data 3

**Description:** Raw values for the METASCAPE pathway enrichment analysis of the identified proteome changes at the organ level.
