# Supplementary material for: Proteomic atlas of organ vasculopathies triggered by Staphylococcus aureus sepsis
Source: Nat Commun. 2019 Oct 11;10:4656. doi: 10.1038/s41467-019-12672-x (PMC6789120; doi:10.1038/s41467-019-12672-x)
Supplement: Supplementary file 7 — Reporting Summary [file 41467_2019_12672_MOESM7_ESM.pdf]

nature research

Corresponding author(s): Jeffrey D Esko

Last updated by author(s): Sep 16, 2019

Reporting Summary

Nature Research wishes to improve the reproducibility of the work that we publish. This form provides structure for consistency and transparency in reporting. For further information on Nature Research policies, see Authors & Referees and the Editorial Policy Checklist.

Statistics

For all statistical analyses, confirm that the following items are present in the figure legend, table legend, main text, or Methods section.

n/a	Confirmed	
☐	☒	The exact sample size (*n*) for each experimental group/condition, given as a discrete number and unit of measurement
☐	☒	A statement on whether measurements were taken from distinct samples or whether the same sample was measured repeatedly
☐	☒	The statistical test(s) used AND whether they are one- or two-sided<br>*Only common tests should be described solely by name; describe more complex techniques in the Methods section.*
☐	☒	A description of all covariates tested
☐	☒	A description of any assumptions or corrections, such as tests of normality and adjustment for multiple comparisons
☐	☒	A full description of the statistical parameters including central tendency (e.g. means) or other basic estimates (e.g. regression coefficient) AND variation (e.g. standard deviation) or associated estimates of uncertainty (e.g. confidence intervals)
☐	☒	For null hypothesis testing, the test statistic (e.g. *F*, *t*, *r*) with confidence intervals, effect sizes, degrees of freedom and *P* value noted<br>*Give P values as exact values whenever suitable.*
☒	☐	For Bayesian analysis, information on the choice of priors and Markov chain Monte Carlo settings
☒	☐	For hierarchical and complex designs, identification of the appropriate level for tests and full reporting of outcomes
☐	☒	Estimates of effect sizes (e.g. Cohen's *d*, Pearson's *r*), indicating how they were calculated

Our web collection on statistics for biologists contains articles on many of the points above.

Software and code

Policy information about availability of computer code

Data collection	Thermo Xcalibur 3.0.63
Data analysis	Maxquant version 1.6.1.0; Perseus version 1.6.1.1; Cytoscape version 3.6.1

For manuscripts utilizing custom algorithms or software that are central to the research but not yet described in published literature, software must be made available to editors/reviewers. We strongly encourage code deposition in a community repository (e.g. GitHub). See the Nature Research guidelines for submitting code & software for further information.

Data

Policy information about availability of data

All manuscripts must include a data availability statement. This statement should provide the following information, where applicable:
- Accession codes, unique identifiers, or web links for publicly available datasets
- A list of figures that have associated raw data
- A description of any restrictions on data availability

The proteomics raw data and metadata have been deposited in the MassIVE archive (PXD013513) and is publicly available.

Field-specific reporting

Please select the one below that is the best fit for your research. If you are not sure, read the appropriate sections before making your selection.

☒ Life sciences ☐ Behavioural & social sciences ☐ Ecological, evolutionary & environmental sciences

For a reference copy of the document with all sections, see nature.com/documents/nr-reporting-summary-flat.pdf

Life sciences study design

All studies must disclose on these points even when the disclosure is negative.

Sample size	Each experiment consisted of three infected and three non-infected mice. One non-infected and one infected mouse were perfused with saline to account for potential background signals. Five different organs were selected for analysis based on their clinical relevance: liver, kidney, heart, brain and white adipose tissue (WAT). To increase throughput and to minimize variability, the work-flow was automated by transferring all sample preparations to a BRAVO liquid handling platform. In total, we performed four independent experiments resulting in 160 LC-MS/MS runs.
Data exclusions	MaxQuant results were imported into Perseus and identified hits were filtered based on number of peptides (>2) and number of MS/MS scans for each peptide (>2)
Replication	Correlation analysis across replicates from the different groups was performed. As described in the result section, there was good agreement across same group replicates.
Randomization	Statistically significant changes between groups were assessed by a two-tailed student's t-test using a permutation-based FDR for multiple test correction and truncation after 250 randomizations
Blinding	Not relevant for the study

Reporting for specific materials, systems and methods

We require information from authors about some types of materials, experimental systems and methods used in many studies. Here, indicate whether each material, system or method listed is relevant to your study. If you are not sure if a list item applies to your research, read the appropriate section before selecting a response.

Materials & experimental systems

n/a	Involved in the study	
☐	☒	Antibodies
☒	☐	Eukaryotic cell lines
☒	☐	Palaeontology
☐	☒	Animals and other organisms
☒	☐	Human research participants
☒	☐	Clinical data

Methods

n/a	Involved in the study	
☒	☐	ChIP-seq
☒	☐	Flow cytometry
☒	☐	MRI-based neuroimaging

Antibodies

Antibodies used	Streptavidin Alexa Fluor 488 (Invitrogen); Isolectin B4 Alexa Fluor 594 (Thermo Fischer Scientific); streptavidin-IRDye680 (LI-COR Biosciences); Lubricin/PRG4 antibody (Novus Biologicals), clone NBP1-19048
Validation	IHC-P, IHC reactivity reported in scientific literature (PMID: 23705804), (23083677).<br><br>Clearfield D, Xin X, Yadav S et al. Osteochondral Differentiation of Fluorescent Multi-Reporter Cells on Zonally-Organized Biomaterials Tissue Eng Part A Aug 23 2018 [PMID: 30136616] (IHC, Mouse) IHC Mouse<br><br>Shepard JB, Jeong JW, Maihle NJ et al. Transient anabolic effects accompany epidermal growth factor receptor signal activation in articular cartilage in vivo. Arthritis Res Ther 2013 May 25 [PMID: 23705804] (IHC-P, Mouse) IHC-P Mouse<br><br>Musumeci G, Loreto C, Carnazza ML et al. Acute injury affects lubricin expression in knee menisci. An immunohistochemical study Ann Anat 2012 Sep 12 [PMID: 23083677] (IHC, Human) IHC Human<br><br>Leonardi R, Musumeci G, Sicurezza E, Loreto C. Lubricin in human temporomandibular joint disc: An immunohistochemical study. Arch Oral Biol. 2012 Jan 11. [PMID: 22244189] (IHC, Human) IHC Human

Animals and other organisms

Policy information about studies involving animals; ARRIVE guidelines recommended for reporting animal research

Laboratory animals	8-10-week-old C57Bl/6 male and female mice
Wild animals	This study did not involve wild animals
Field-collected samples	This study did not involve samples collected from the field

Ethics oversight	Animals were housed and bred in vivaria approved by the Association for Assessment and Accreditation of Laboratory Animal Care located in the School of Medicine, UC San Diego. All experiments were performed in accordance with relevant guidelines and regulations following standards and procedures approved by the UC San Diego Institutional Animal Care and Use Committee.

Note that full information on the approval of the study protocol must also be provided in the manuscript.
